# Significance of MEF2C and RUNX3 Regulation for Endochondral Differentiation of Human Mesenchymal Progenitor Cells

**DOI:** 10.3389/fcell.2020.00081

**Published:** 2020-03-04

**Authors:** Simon I. Dreher, Jennifer Fischer, Tilman Walker, Solvig Diederichs, Wiltrud Richter

**Affiliations:** ^1^Research Center for Experimental Orthopaedics, Heidelberg University Hospital, Heidelberg, Germany; ^2^Clinic for Orthopaedics and Trauma Surgery, Heidelberg University Hospital, Heidelberg, Germany

**Keywords:** chondrogenesis, cartilage, hypertrophy, RUNX2, FGF, WNT, BMP, PTHrP

## Abstract

Guiding progenitor cell development between chondral versus endochondral pathways is still an unachieved task of cartilage neogenesis, and human mesenchymal progenitor cell (MPC) chondrogenesis is considered as a valuable model to better understand hypertrophic development of chondrocytes. Transcription factors Runx2, Runx3, and Mef2c play prominent roles for chondrocyte hypertrophy during mouse development, but little is known on the importance of these key fate-determining factors for endochondral development of human MPCs. The aim of this study was to unravel the regulation of RUNX2, RUNX3, and MEF2C during MPC chondrogenesis, the pathways driving their expression, and the downstream hypertrophic targets affected by their regulation. *RUNX2*, *RUNX3*, and *MEF2C* gene expression was differentially regulated during chondrogenesis of MPCs, but remained low and unregulated when non-hypertrophic articular chondrocytes were differentiated under the same conditions. RUNX3 and MEF2C mRNA and protein levels rose in parallel to hypertrophic marker upregulation, but surprisingly, *RUNX2* gene expression changed only by trend and RUNX2 protein remained undetectable. While *RUNX3* expression was driven by TGF-β and BMP signaling, *MEF2C* responded to WNT-, BMP-, and Hedgehog-pathway inhibition. *MEF2C* but not *RUNX3* levels correlated significantly with *COL10A1*, *IHH*, and *IBSP* gene expression when hypertrophy was attenuated. *IBSP* was a downstream target of RUNX3 and MEF2C but not RUNX2 in SAOS-2 cells, underlining the capacity of RUNX3 and MEF2C to stimulate osteogenic marker expression in human cells. Conclusively, RUNX3 and MEF2C appeared more important than RUNX2 for human endochondral MPC chondrogenesis. Pathways altering the speed of chondrogenesis (FGF, TGF-β, BMP) affected RUNX2 or RUNX3, while pathways changing hypertrophy (WNT, PTHrP/HH) regulated mainly MEF2C. Taken together, reduction of MEF2C levels is a new goal to shift human cartilage neogenesis toward the chondral pathway.

## Introduction

Joint surface defects often caused by traumatic injury are a common cause for development of osteoarthritis (OA) (Buckwalter and Mankin, 1997). However, the restricted availability of human articular chondrocytes (ACs), as well as a rather limited understanding of articular cartilage neogenesis, still hampers the development of successful therapies to restore joint surface defects. A promising alternative cell source for articular chondrocytes are bone marrow-derived mesenchymal progenitor cells (MPCs), formerly referred to as mesenchymal stromal cells (MSCs), owing to their high proliferation and differentiation potential. *In vitro* generation of cartilage tissue from MPCs is realized by defined *in vitro* settings and verified by detection of collagen type II and proteoglycan deposition (Yoo et al., 1998; Winter et al., 2003). Unfortunately, however, MPC *in vitro* chondrogenesis is hampered by undesired intrinsic endochondral commitment manifested by a hypertrophic phenotype. MPC hypertrophy is typically associated with upregulation of collagen type X, bone sialoprotein (IBSP), alkaline phosphatase (ALP), and the resulting propensity of the formed tissue to degenerate and undergo mineralization *in vivo* (Pelttari et al., 2006; Weiss et al., 2010). Instead, human ACs redifferentiate without signs of hypertrophy and produce permanent cartilage, which does not mineralize *in vivo* (Pelttari et al., 2006). Re-directing MPC chondrogenesis to obtain stable chondrocytes is an active field of investigation, but the specific drivers for hypertrophic and osteogenic marker upregulation during MPC chondrogenesis remain currently unknown.

Transcription factors are master regulators of cell differentiation, and several transcription factors are held responsible for hypertrophic development of chondrocytes during embryonic development of transient cartilage in mice (Lefebvre and Bhattaram, 2010; Studer et al., 2012). The Runt family transcription factors Runx2 and Runx3 are expressed in prehypertrophic and hypertrophic chondrocytes (Inada et al., 1999; Kim et al., 1999; Enomoto-Iwamoto et al., 2001; Choi et al., 2002) and appear highly relevant for endochondral differentiation. Runx2-deficient mice lack prehypertrophic and hypertrophic chondrocytes in several locations, all endochondral bones are cartilaginous, and mineralization is mostly absent throughout the skeleton, except tibia, fibula, radius, and ulna (Komori et al., 1997; Inada et al., 1999; Yoshida et al., 2004). Forced expression of *Runx2* in murine immature chondrocytes drives premature maturation and induces the expression of *Col10a1* and other hypertrophic markers *in vivo* (Takeda et al., 2001; Ueta et al., 2001; Stricker et al., 2002), as well as in immature chondrocytes from chicken sternum *in vitro* (Enomoto et al., 2000). Conversely, genetic ablation, or the expression of dominant negative *Runx2* in mice, results in delayed or diminished chondrocyte hypertrophy (Inada et al., 1999; Kim et al., 1999; Ueta et al., 2001). However, hypertrophy was found to be completely absent only in *Runx2* and *Runx3* double knockout mice (Yoshida et al., 2004). Since chondrocyte maturation was only slightly retarded in *Runx3*-deficient mice (Yoshida et al., 2004), Runx2 is generally regarded as the dominant regulator of chondrocyte hypertrophy (Komori, 2015; Nishimura et al., 2017, 2018).

Another important transcription factor critical for the early stage hypertrophy of chondrocytes is MEF2C, a member of the myocyte enhancer factor 2 (MEF2) family of transcription factors (Arnold et al., 2007). Chondrocyte-specific *Mef2c*-deficient mice display shortening of the bones, suppression of chondrocyte hypertrophy, and endochondral ossification (Arnold et al., 2007), while gain of function experiments in chondrocytes result in premature and excessive endochondral ossification (Arnold et al., 2007). Mef2c was indicated to act upstream of Runx2, necessary to either induce or maintain Runx2 expression in hypertrophic chondrocytes and to regulate *Col10a1* (Arnold et al., 2007). *In vitro* analyses with a *Col10a1* promoter driven luciferase plasmid in COS7 cells demonstrated direct regulation of *Col10a1* expression by Mef2c (Arnold et al., 2007).

In spite of the prominent role of Runx2, Runx3, and Mef2c for chondrocyte hypertrophy during mouse development and in cell lines of various species, surprisingly little is currently known about regulation of these key fate-determining factors in human chondrocyte development and during chondrogenesis of human MPCs. So far, RUNX2, RUNX3, and MEF2C regulation and their involvement in endochondral MPC differentiation have neither been studied thoroughly on mRNA nor on protein level, and the transcription factors driving hypertrophic development during *in vitro* chondrogenesis of MPCs remain unclear.

The aim of this study was to uncover the regulation of RUNX2, RUNX3, and MEF2C transcription factors during endochondral MPC chondrogenesis in comparison to chondral AC redifferentiation and to assess which signaling pathways drive their expression during MPC chondrogenesis and which hypertrophic markers may be targeted by these transcription factors.

Gene expression and protein levels of RUNX2, RUNX3, and MEF2C were assessed before and during *in vitro* chondrogenesis of MPCs, and levels were compared to redifferentiating ACs. TGF-β, BMP, FGF, WNT, and PTHrP/HH signaling, which are known to affect differentiation and hypertrophy of MPCs, were manipulated by removal of agonists or addition of antagonists to unravel their contribution to RUNX2, RUNX3, and MEF2C regulation. Knockdown of RUNX/MEF2C protein levels by siRNA was performed to understand their role for hypertrophic marker expression. Better knowledge of hypertrophy-associated transcription factor regulation may enable optimal guiding of MPCs between chondral versus endochondral pathways to improve cell-based cartilage and bone regeneration strategies.

## Materials and Methods

### Isolation and Expansion of MPCs and ACs

Fresh bone marrow aspirates from 12 female and 10 male patients, age 16–77 (mean 56) years, undergoing total hip replacement surgery and samples of human articular cartilage from 3 female and 2 male patients, age 57–79 (mean 70), years undergoing total knee replacement surgery were acquired with informed written consent of the patients. Samples were obtained after approval by the ethics committee on human experimentation of the Medical Faculty of Heidelberg University and in agreement with the Helsinki Declaration of 1975 in its latest version. MPCs were isolated, as described (Winter et al., 2003). Briefly, samples were subjected to Ficoll Paque Plus density gradient centrifugation. The mononuclear cell fraction was seeded in expansion medium consisting of high-glucose Dulbecco’s modified Eagle’s medium (DMEM), 12.5% fetal calf serum (FCS), 2 mM L-glutamine, 1% non-essential amino acids, 0.1% β-mercaptoethanol (all from Gibco, Life Technologies, Germany), 1% penicillin/streptomycin (Biochrom, Germany), and 4 ng/mL of recombinant basic fibroblast growth factor (Miltenyi Biotec, Germany). Cells were passaged at 5 × 10^3^ cells/cm^2^ up to passage 3. ACs were isolated as previously described (Praxenthaler et al., 2017). Cartilage from regions with no evident degeneration was harvested, minced, and digested overnight with 1.5 mg/mL of collagenase B (Roche Diagnostics, Germany) and 0.1 mg/mL of hyaluronidase (Sigma Aldrich, Germany). Washed chondrocytes were plated at 5 × 10^3^ cells/cm^2^ and expanded for two passages in low-glucose DMEM supplemented with 10% FCS, 1% penicillin/streptomycin.

### Chondrogenic (Re-)differentiation

MPCs and ACs were cultured as 3D pellets (5 × 10^5^ cells/pellet) in chondrogenic medium (DMEM high-glucose; Gibco, Life Technologies, Germany), 0.1 μM dexamethasone, 0.17 mM ascorbic acid-2 phosphate, 5 μg/mL transferrin, 5 ng/mL selenous acid, 2 mM sodium pyruvate, 0.35 mM proline, 1.25 mg/mL bovine serum albumin (all from Sigma Aldrich, Germany), 5 μg/mL insulin (Lantus, Sanofi-Aventis, Germany), or ITS + premix (Corning, Germany), 1% penicillin/streptomycin (Biochrom, Germany), 10 ng/mL recombinant human TGF-β1 (PeproTech or Miltenyi, Germany) for up to 6 weeks at 37°C, 6% CO_2_ with medium changes three times a week. For indicated time points, chondrogenic medium was supplemented with the BMP-inhibitor dorsomorphin (10 μM in 0.1% DMSO, day 14–42; ENZO Life Science, United States), the WNT-inhibitor IWP-2 (2 μM in 0.04% DMSO, day 14–35; Tocris Bioscience, United Kingdom), the FGF-inhibitor PD173074 (250 nM in 0.02% DMSO, day 7–42; Sigma Aldrich, Germany), or the respective solvent. Where indicated, TGF-β was withdrawn from day 21 onward. One group received daily PTHrP(1-34) treatment for 6 h (2.5 nM in water; Bachem, Germany) as described before (Fischer et al., 2016).

### siRNA-Mediated Knockdown in SAOS-2

SAOS-2 cells were cultured in DMEM high-glucose, 10% FCS, 1% penicillin/streptomycin. Cells (1 × 10^6^) were electroporated in presence of 0.5 nmol siCtrl (SIC001), siMEF2C (SASI Hs01 00233876), siRUNX2 (SASI Hs01 00072700), or siRUNX2 (SASI Hs01 00220066) (all from Sigma Aldrich, Germany), with two 15 ms pulses of 1300 V (MicroPorator, PEQlab/VWR, Germany, and Neon Transfection System, Thermo Fischer, Germany). Cells were seeded at 3 × 10^4^ cells/cm^2^ in culture medium without penicillin/streptomycin for 1 day. Analyses were performed 48 h after transfection.

### Quantitative RT-PCR (qRT-PCR)

Total RNA was extracted from three pooled pellets per donor, group, and time point. After guanidinium isothiocycante/phenol extraction (peqGOLD Trifast, Peqlab, Germany), oligo(dT)-coupled magnetic beads (Dynabeads, Life Technologies, Germany) were used to extract polyadenylated mRNA, which was then reverse transcribed into cDNA using the reverse transcriptase Omniscript (Qiagen, Germany), and oligo(dT) primers. The expression level of individual genes was determined by quantitative real time PCR (qPCR; Roche Diagnostics, Germany, or Stratagen, United States), primer pairs used for amplification are listed in Table 1. Genes were rated as expressed when gel electrophoresis of PCR amplificates showed a clear band of the correct size. Gene expression levels were calculated using the ΔCt method with arithmetic mean expression of reference genes *CPSF6* and *HNRPH1*. The ΔCt was calculated by subtracting the mean reference Ct from the Ct value of the gene of interest. Indicated % reference gene (%RefG) was calculated as percentage of 1.8^(−ΔCt).

**TABLE 1:** Primer pairs for qRT-PCR analysis in alphabetical order.

**Gene**	**Forward**	**Reverse**
*ACAN*	5′-GGAACCACTTGGGTCACG-3′	5′-GCACATGCCTTCTGCTT-3′
*ALPL*	5′-CACCAACGTGGCTAAGAATG-3′	5′-ATCTCCAGCCTGGTCTCCTC-3′
*COL2A1*	5′-TGGCCTGAGACAGCATGAC-3′	5′-AGTGTTGGGAGCCAGATTGT-3′
*COL10A1*	5′-TTTACGCTGAACGATACCAAA-3′	5′-TTGCTCTCCTCTTACTGCTAT-3′
*CPSF6*	5′-AAGATTGCCTTCATGGAATTGAG-3′	5′-TCGTGATCTACTATGGTCCCTCTCT-3′
*GLI1*	5′-TGCAGTAAAGCCTTCAGCAATG-3′	5′-TTTTCGCAGCGAGCTAGGAT-3′
*HNRPH1*	5′-GATGTAGCAAGGAAGAAATTGTTCAG-3′	5′-CACCGGCAATGTTATCCCAT-3′
*IBSP*	5′-CAGGGCAGTAGTGACTCATCC-3′	5′- TCGATTCTTCATTGTTTTCTCCT -3′
*IHH*	5′-CGACCGCAATAAGTATGGAC-3′	5′-GGTGAGCGGGTGTGAGTG-3′
*MEF2C*	5′-GTATGGCAATCCCCGAAACT-3′	5′-ATCGTATTCTTGCTGCCTGG-3′
*RUNX2*	5′-ACTCTACCACCCCGCTGTC-3′	5′-CAGAGGTGGCAGTGTCATCA-3′
*RUNX3*	5′-CAAGATGGGCGAGAACAGC-3′	5′-ATCACAGTCACCACCGTACC-3′
*SOX9*	5′-GTACCCGCACTTGCACAAC-3′	5′-TCGCTCTCGTTCAGAAGTCTC-3′
*SPP1*	5′- GCTAAACCCTGACCCATCTC -3′	5′- ATAACTGTCCTTCCCACGGC-3′

### ALP Activity

Culture supernatants conditioned for 2 days were collected and pooled, and 100 μL conditioned media were incubated with 100 μL of substrate solution (10 mg/mL p-nitrophenylphosphate in 0.1 M glycine; Carl Roth, Germany), 1 mM MgCl_2_, and 1 mM ZnCl_2_ (all from Sigma Aldrich, Germany), pH 9.6. Absorbance was recorded at 405/490 nm (Sunrise, Tecan, Switzerland); enzyme activity was referred to a p-nitrophenol-derived standard curve (Sigma Aldrich, Germany) and calculated as ALP activity (ng/mL/min).

### Western Blotting

Two to three pellets were pooled in 150 mL PhosphoSafe Extraction Reagent (Merck Millipore, Germany) supplemented with 1 mM Pefabloc1 SC (Sigma Aldrich, Germany), minced in a mixer mill (Retsch, Germany) at 30 Hz and centrifuged at 13,000 × *g* for 20 min to remove cellular debris. Samples were separated by denaturing sodium-dodecyl sulfate polyacrylamid gel electrophoresis, and blotted on nitrocellulose (GE Healthcare, Amersham, Germany). To probe for several proteins of interest in the same samples, the membrane was cut horizontally at 50 kDa. The membranes were probed with mouse anti-β-actin antibody (clone AC-15; 1:10,000; GeneTex; GTX26276), rabbit polyclonal anti-SOX9 antibody (1:2,000; Merck Millipore; AB5535), rabbit monoclonal anti-MEF2C antibody (clone D80C1, 1:2,000; Cell Signaling Technology; 5030), mouse monoclonal anti-RUNX2 antibody (clone 8G5; 1:1,000; MBL; D130-3), mouse monoclonal anti-RUNX3 antibody (clone R3-5G4; 1:200; Merck Millipore; MABE145), mouse monoclonal anti-pERK antibody (clone E-4; 1:200; Santa Cruz; sc-7383), or rabbit polyclonal anti-EKR1/2 antibody (1:1,000; Cell Signaling Technology; 9102). Bands were detected by peroxidase-coupled goat anti-mouse antibody (1:5,000; Jackson ImmunoResearch Laboratories) or peroxidase-coupled goat anti-rabbit antibody (1:10,000; Jackson ImmunoResearch Laboratories) and visualized by enhanced chemiluminescence (Roche Diagnostics, Germany).

## Histology

Pellets were fixed for 2 h in 4% formaldehyde, dehydrated in a graded isopropanol series, and paraffin embedded. To visualize deposited proteoglycans, 5-μm sections were deparaffinized and rehydrated and stained with 0.2% (w/v) Safranin O (Fluka, Sigma Aldrich, Germany) in 1% acetic acid, using Certistain Fast Green [Merck, Germany, 0.04% (w/v) in 0.2% acetic acid] as counterstain, following standard protocols. Immunohistology was performed as described (Winter et al., 2003). Briefly, 5-μm sections were treated with 4 mg/mL hyaluronidase in PBS, pH 5.5, and 1 mg/mL pronase (both from Roche Diagnostics, Germany), blocked with 5% BSA (Sigma Aldrich, Germany) and stained with anti-human collagen type II antibody (1:1,000, clone II-4C11; ICN Biomedicals, Germany) or with a mouse anti-human collagen type X antibody (X53; Quartett, Germany), followed by biotinylated goat anti-mouse antibody (1:500, Dianova, Germany) and streptavidine alkaline-phosphatase fast red (Roche Diagnostics). For Aggrecan staining, targets were retrieved with 4 mg/mL hyaluronidase in PBS, pH 5.5, and 0.02 mg/ml proteinase XXIV in PBS, pH 7.4. As primary antibody mouse monoclonal anti-aggrecan (1:25 in 1% BSA; SM1353S; Acris; Germany), followed by polyclonal AP anti-mouse IgG and ImmPACT Vector Red alkaline-phosphatase substrate (VECTOR, United States), was used. For MEF2C target retrieval, sections were boiled in 1 mM EDTA, pH 9, in aqua dest. Endogenous peroxidase activity was blocked with 3% H_2_O_2_ in TBS-T, and unspecific binding sites were blocked with 1% BSA in TBS-T. Rabbit monoclonal anti-MEF2C antibody (1:500 in 1% BSA in TBS-T; abcam; Netherlands), was followed by a polyclonal HRP-conjugated anti-Rb IgG antibody and DAB staining. For RUNX2 and RUNX3 staining sections were boiled in Citrate Target Retrieval Solution, pH 6 (1:10 in aqua dest.; Dako; Denmark). Mouse monoclonal anti-RUNX2 (1:100 in 1% BSA in TBS-T; clone 8G5; D130-3; MBL Biozol; Germany) or mouse monoclonal anti-RUNX3 (1:400 in 1% BSA in TBS-T; clone D9K6L; 13089; Cell Signaling Technology; United States), followed by mouse-specific HRP/DAB (ABC) Detection IHC Kit (ab64259; abcam; Amsterdam, Netherlands) were used. Nuclei were counterstained with Mayer’s Hematoxylin. ALP activity was detected via an NBT/BCIP conversion (2% in phosphate saline buffer; Roche).

## Statistics

Results are shown as median values and are depicted as boxplots, with each box representing the interquartile range (IQR) extending between the 25th and 75th percentiles, and lines inside the boxes represent the median. Whiskers extend to minimum and maximum values, outliers (between 1.5 × IQR and 3 × IQR) are depicted as ∘, and extreme values (beyond 3 × IQR) as □. Statistical significance between two groups was calculated using Mann–Whitney *U* test. The *p* value was adjusted for multiple comparisons using Bonferroni correction. For time-courses, data are expressed as the mean ± standard deviation. ANOVA with Tukey *post hoc* test was used for multiple comparisons within one time-course. A probability value of *p* ≤ 0.05 was considered statistically significant. Correlations were determined recording the Pearson’s coefficient. All statistical tests were calculated with SPSS 25.0 (IBM, Germany).

## Results

### Transcription Factor Signature in Expanded MPCs and ACs

**FIGURE 1:**
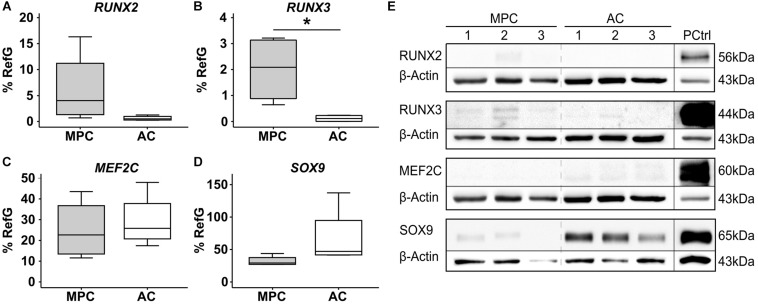
Transcription factor signature of expanded MPCs. MPCs and ACs were harvested at passages 2–3 of expansion (*n* = 4 donors). **(A)** *RUNX2*, **(B)** *RUNX3*, **(C)** *MEF2C*, and **(D)** *SOX9* mRNA levels were determined by qPCR and levels were referred to reference genes *HNRPH1* and *CPSF6*. Data are shown as box plots, with each box representing the interquartile range (IQR) extending between the 25th and 75th percentiles, and lines inside the boxes representing the median. Whiskers extend to minimum and maximum values. *, *p* ≤ 0.05, Mann–Whitney *U* test. **(E)** RUNX2, RUNX3, MEF2C, and SOX9 protein levels were determined by Western blot analysis in cell lysates from three donors (MPC1-3, AC1-3) on the same gel, β-actin levels were determined as internal reference, and SAOS-2 cell lysate was used as positive control on the same gel (PCtrl).

Mesenchymal progenitor cells were characterized after expansion according common criteria (Dominici et al., 2006), and expression of typical surface markers, as well as differentiation into the adipogenic, osteogenic, and chondrogenic lineage, was confirmed (data not shown). First, we tested whether *RUNX2*, *RUNX3*, or *MEF2C* were differentially expressed in expanded MPC and AC populations before start of differentiation. Significantly higher *RUNX3* levels (*p* = 0.029), and a trend for elevated *RUNX2* expression (*p* = 0.057) in MPCs, argued in favor of a pro-osteogenic signature of expanded MPCs versus ACs (Figures 1A,B), while similar expression was seen for *MEF2C* (Figure 1C). Little differences were evident on protein levels where RUNX2 remained below and RUNX3 close to detection limit in MPCs in Western blots (Figure 1E). As expected, MPCs showed lower mean *SOX9* mRNA expression (Figure 1D) and strikingly lower SOX9 protein levels, in line with the lower chondrogenic commitment of progenitor cells compared to ACs (Figure 1E). Thus, main differences between expanded MPCs and ACs at the protein level for tested transcription factors occurred only for SOX9.

### Upregulation of RUNX3 and MEF2C With Hypertrophy During MPC Chondrogenesis

**FIGURE 2:**
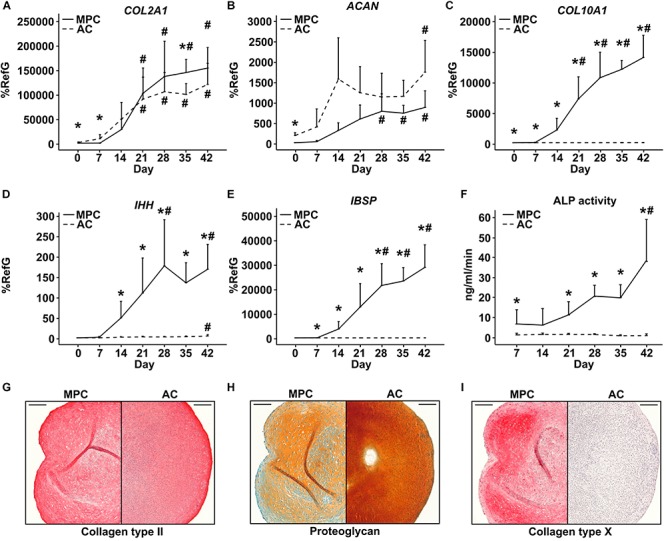
Characterization of MPC and AC differentiation. MPC and AC pellets were subjected to chondrogenic induction for 6 weeks. Samples harvested in weekly intervals were subjected to gene expression analysis by qPCR for indicated genes **(A–E)**, and levels were referred to reference genes *HNRPH1* and *CPSF6* (*n* = 4 donors). **(F)** Alkaline phosphatase (ALP) activity was determined in culture supernatants pooled from five pellets (*n* = 4 donors). Data are shown as mean ± standard deviation over the time course of 6 weeks. MPC versus AC; Mann–Whitney *U* test; *, *p* ≤ 0.05 versus d0 (d7 ALP activity) ANOVA Tukey; #, *p* ≤ 0.05. **(G–I)**: Paraffin sections of AC and MPC pellets at day 42 were stained for **(G)** collagen type II and **(I)** collagen type X by immunohistochemistry or with **(H)** Safranin O/Fast Green to visualize proteoglycan deposition. Scale bars represent 200 μm.

Next, MPCs were subjected to chondrogenic induction for 6 weeks, and ACs were cultured, as controls, under identical conditions to allow redifferentiation. Both cell types (re-) differentiated according to strong elevation of *COL2A1* (Figure 2A) and *ACAN* expression (Figure 2B) and deposited collagen type II (Figure 2G) as well as proteoglycans (Figure 2H) in the extracellular matrix. However, only MPCs strongly upregulated the hypertrophic markers *COL10A1* (Figure 2C), *IHH* (Figure 2D), and *IBSP* (Figure 2E); showed rising ALP activity (Figure 2F); and accumulated collagen type X in pellets (Figure 2I). This confirmed endochondral differentiation exclusively in MPCs.

**FIGURE 3:**
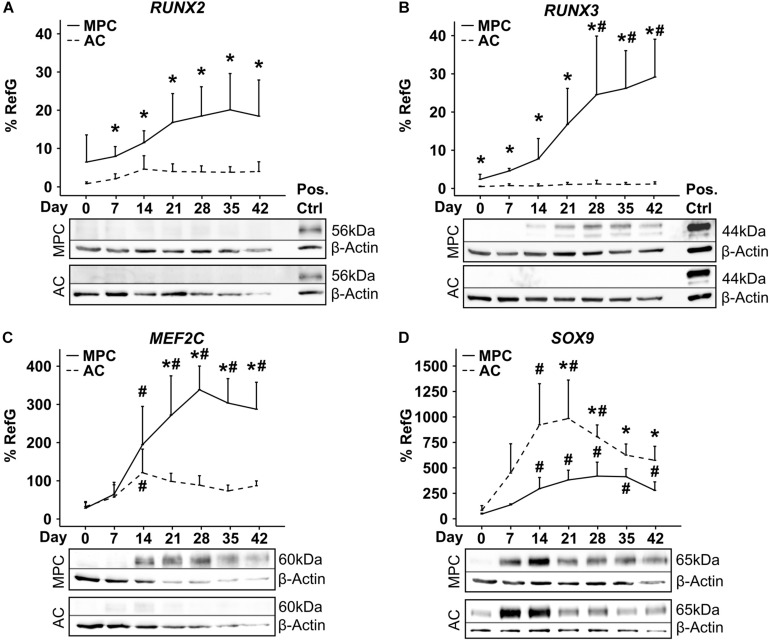
Transcription factor regulation during MPC chondrogenesis versus AC redifferentiation. MPC and AC pellets were subjected to chondrogenic induction for 6 weeks. Samples were harvested in weekly intervals, and transcription factor levels were determined on mRNA and protein levels (*n* = 4 donors). Indicated gene expression levels were determined by qPCR **(A–D)** and referred to reference genes *HNRPH1* and *CPSF6*. Data show mean ± standard deviation. MPC versus AC, Mann–Whitney *U* test; *, *p* ≤ 0.05 versus d0 ANOVA Tukey; #, *p* ≤ 0.05. RUNX2, RUNX3, MEF2C, and SOX9 protein levels were determined by Western blot analysis, β-actin levels were determined as internal reference, and SAOS-2 cell lysate was used as positive control on the same gel (Pos. Ctrl). Shown is one representative of three to four experiments.

In spite of pronounced hypertrophic differentiation of MPCs, *RUNX2* gene expression did not increase significantly compared to day 0 (Figure 3A). Although *RUNX2* mRNA was significantly higher than in corresponding AC samples from day 7 onward, RUNX2 protein remained below detection limit during the entire MPC chondrogenesis by Western blotting (Figure 3A). In contrast, *RUNX3* mRNA rose strongly in MPCs parallel to hypertrophic markers, reached significantly higher levels from day 28 on, and was significantly above AC levels at each timepoint (Figure 3B). In line, RUNX3 protein was strongly upregulated during MPC chondrogenesis while remaining absent during AC redifferentiation. MEF2C mRNA and protein levels were regulated parallel to RUNX3, with significantly higher expression in MPCs from day 21 on when hypertrophy was overt (Figure 3C). SOX9 mRNA and protein regulation was similar in MPCs and ACs, but levels peaked earlier in ACs (Figure 3D). When protein levels were compared on the same blot, SOX9 was considerably stronger in AC versus MPC cultures at all timepoints as expected (Supplementary Figure S1).

Histological staining of aggrecan, ALP activity and of transcription factors MEF2C, RUNX3, and RUNX2 over time showed a similar deposition of ECM molecules in MPC- and AC-derived cartilage (Supplementary Figure S2A) but a strong MEF2C as well as RUNX3 staining on day 28 and 42 in nuclei of MPC-derived chondrocytes located in the periphery of pellets (Supplementary Figure S2B). Cells in AC pellets never turned positive at any time point. In contrast, very weak nuclear RUNX2 staining was obvious on day 42 in some peripheral MPC-derived chondrocytes. Strong staining of SAOS-2 cells embedded in fibrin hydrogel to allow identical processing, confirmed a positive MEF2C, RUNX3, and RUNX2 staining (Supplementary Figure S2C). This underlined the main findings of Western blots and demonstrated that mainly peripheral MPCs underwent hypertrophy, which is in line with peripheral detection of ALP activity (Supplementary Figure S2A) reported before (Dickhut et al., 2009; Fischer et al., 2010; Mueller et al., 2013; Diederichs et al., 2017). In conclusion, specific microenvironmental conditions like higher oxygen and/or better nutrient access were necessary to drive cells into hypertrophy while chondrocytes in the center developed properly.

Taken together, RUNX3 and MEF2C proteins accumulated parallel to hypertrophy only in MPC pellets and in the virtual absence of RUNX2 protein, its importance for endochondral MPC commitment remained elusive.

### RUNX3 Expression During MPC Chondrogenesis Is TGF-β-Dependent

**FIGURE 4:**
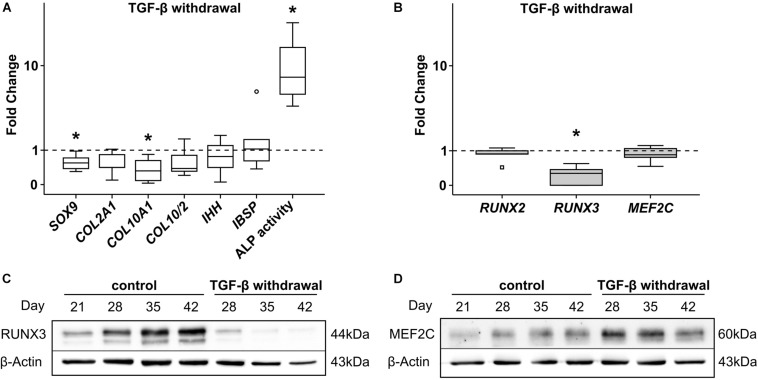
Effects of TGF-β withdrawal on RUNX2, RUNX3, and MEF2C. MPC pellets were subjected to chondrogenic induction for 3 weeks before TGF-β was omitted from chondrogenic medium for half of the pellets, while other ingredients remained constant until week 6 (*n* = 5 donors). Expression of indicated genes was determined at day 42 by qPCR **(A,B)**, and levels were referred to reference genes *HNRPH1* and *CPSF6*. ALP activity was quantified in culture supernatants (*n* = 4 donors) **(A)**. Expression levels of indicated genes and ALP activity are referred to control cultures (dashed lines). Box plots were generated as described in Figure 1, outliers are depicted as ∘, and extreme values as □. *, *p* ≤ 0.05, Mann–Whitney *U* test compared to control. **(C)** RUNX3 and **(D)** MEF2C protein levels were determined by Western blot analysis, β-actin levels were determined as internal reference. One representative of three experiments is shown.

In order to unravel which signaling pathways may drive mRNA induction of *RUNX2*, *RUNX3*, and *MEF2C* exclusively in MPCs, multiple pathways were manipulated by removal of agonists or addition of antagonists during MPC chondrogenesis. First, TGF-β was withdrawn from chondrogenic culture medium from day 21 on when hypertrophic markers had risen but not yet reached a plateau. As a result, mean *SOX9*, *COL2A1*, and *COL10A1* gene expression (−42% *SOX9*; −44% *COL2A1*, and −62% *COL10A1*) remained lower at day 42 (Figure 4A) and chondrogenic differentiation was halted. However, *IBSP* was unaffected and ALP enzyme activity was significantly boosted in the absence of TGF-β (Figure 4A), in line with known suppression of mineralization activity by TGF-β (Talley-Ronsholdt et al., 1995; Sowa et al., 2002). Along with diminished differentiation markers, *RUNX3* gene expression was significantly lower after TGF-β withdrawal. In contrast, *MEF2C* as well as *RUNX2* mRNA levels remained unaffected (Figure 4B). In line, RUNX3 protein was almost lost in the absence of TGF-β (Figure 4C) demonstrating that its expression was mainly driven by TGF-β. In contrast, MEF2C protein was maintained or slightly enhanced suggesting its independence of the TGF-β pathway (Figure 4D). Conclusively, TGF-β signaling contributed mainly to *RUNX3* mRNA upregulation and RUNX3 protein accumulation during MPC chondrogenesis, and mineralization-relevant ALP activity was boosted when RUNX3 protein levels dropped.

### Upregulation of RUNX3 and MEF2C, but Not RUNX2, Is BMP-Dependent

**FIGURE 5:**
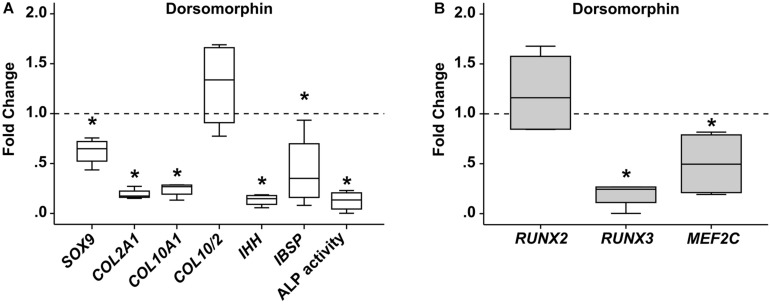
Effects of BMP-receptor inhibition on RUNX2, RUNX3, and MEF2C. MPC pellets were subjected to chondrogenic induction for 2 weeks before treatment with 10 μM dorsomorphin or 0.1% DMSO until week 6 (*n* = 4 donors). Expression of indicated genes was determined by qPCR **(A,B)**, and levels were referred to reference genes *HNRPH1* and *CPSF6*. ALP activity was quantified in culture supernatants **(A)**. Expression levels of indicated genes and ALP activity are referred to DMSO cultures (dashed lines). Box plots were generated as described in Figure 1. *, *p* ≤ 0.05, Mann–Whitney *U* test compared to control.

Next, chondrogenic MPC cultures were treated from day 14 on with the small molecule BMP-receptor inhibitor dorsomorphin (DM) capable of blocking pSmad1/5/9-signaling. Treatment of MPCs with DM from day 14 on is known to reduce chondrogenesis, since less collagen type II protein is produced and also less collagen type X accumulates (Dexheimer et al., 2016). In line, day-42 pellets showed less *SOX9* (−38%) and less *COL2A1* (−81%) gene expression, but also less hypertrophic *COL10A1* (−76%), leaving the *COL10/COL2* ratio unchanged (Figure 5A). As expected at reduced chondrogenesis, also *IHH* and *IBSP* expression was significantly reduced, as was ALP activity in culture supernatants. Importantly, *RUNX2* and *RUNX3* gene expression was differentially regulated under DM. While *RUNX2* gene expression increased by trend compared to DMSO treated control cultures (Figure 5B), *RUNX3* levels were strongly reduced when BMP-signaling was inhibited (Figure 5B). *MEF2C* expression was also significantly lower, demonstrating that Smad1/5/9-signals strongly contributed to the upregulation of *RUNX3* and *MEF2C* during MPC chondrogenesis, but had little effect on regulation of *RUNX2*.

### No Influence of FGF-Receptor Signaling on RUNX3 and MEF2C Levels

**FIGURE 6:**
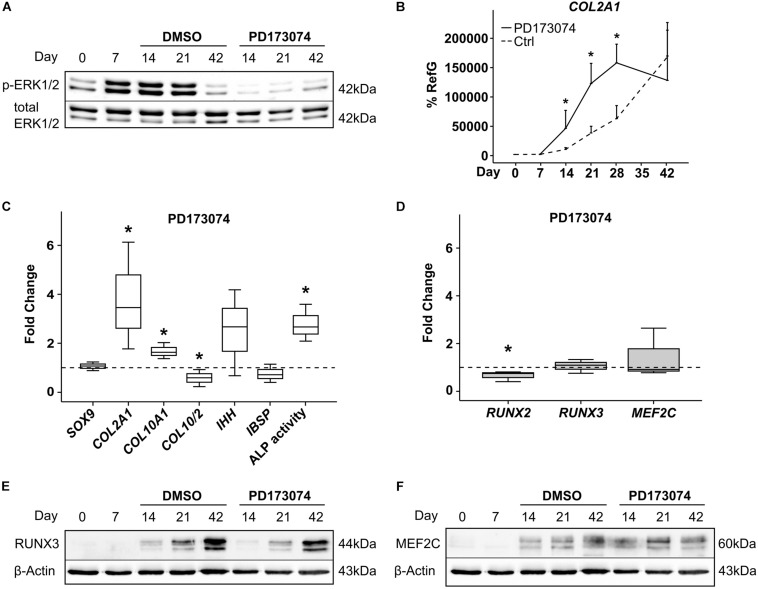
Effects of FGF-receptor inhibition on RUNX2, RUNX3, and MEF2C. MPC pellets were subjected to chondrogenic induction for 6 weeks and treated with 250 nM of the FGFR-Inhibitor PD173074 or with the corresponding amount of DMSO from day 7 onward. Protein levels of pERK1/2 and total ERK1/2 as reference were detected by Western blot **(A)**. Shown is one representative of three experiments. **(B)** Gene expression of *COL2A1* was determined during 42 days of chondrogenesis with PD173074 (black line) or DMSO control (dashed line) (*n* = 3 donors), **(C,D)** expression of indicated genes was determined by qPCR at day 21 of chondrogenesis, and levels were referred to reference genes *HNRPH1* and *CPSF6*. ALP activity was quantified in culture supernatants **(C)**. Expression levels of indicated genes and ALP activity are referred to DMSO cultures (dashed lines). Box plots were generated as described in Figure 1. *, *p* ≤ 0.05, Mann–Whitney *U* test compared to control. **(E,F)** RUNX3 and MEF2C protein levels were determined in samples with DMSO or PD173074 at indicated timepoints, β-actin levels were determined as internal reference. One representative of three experiments is shown.

To challenge a contribution of FGF-signaling to MPC hypertrophy as indicated by Frisch et al. (2016), the FGF receptor (FGFR) inhibitor PD173074 was added to the chondrogenic medium from day 7 of chondrogenesis. FGFR inhibition reduced phospho-ERK1/2 protein levels in MPC pellets on day 14 and day 21 of chondrogenesis, confirming inhibitor activity (Figure 6A). Interestingly, this accelerated the upregulation of the chondrogenic marker *COL2A1* significantly, which reached its peak height already at day 28, compared to day 42 in the controls (Figure 6B). Alongside this, at day 21, *COL10A1* expression and the mineralization-relevant ALP activity were significantly elevated, and IHH rose by trend, indicating an overall faster progression of chondrogenesis of which the osteogenic marker *IBSP* was, however, unaffected (Figure 6C). While *RUNX3* and *MEF2C* expression remained unaltered, *RUNX2* mRNA levels were slightly but significantly reduced, suggesting its expression is driven by FGFR signaling (Figure 6D). Western blotting confirmed unaltered RUNX3 and MEF2C protein levels under FGFR inhibition (Figures 6E,F). Overall, this indicated some active FGFR signaling in chondrogenic cultures, which decelerated chondrogenic differentiation. Opposite to RUNX2, RUNX3, and MEF2C levels were independent of this pathway.

### MEF2C Regulation Is WNT-Dependent

**FIGURE 7:**
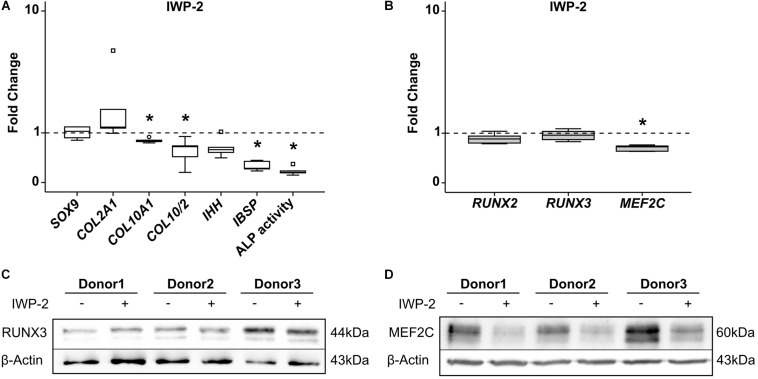
Effects of WNT-signaling inhibition on RUNX2, RUNX3, and MEF2C. MPC pellets were subjected to chondrogenic induction for 5 weeks and treated with 2 μM IWP-2 or the corresponding amount of DMSO from day 14 (*n* = 5 donors). Expression of indicated genes was determined by qRT-PCR **(A,B)**, and levels were referred to reference genes *HNRPH1* and *CPSF6*. ALP activity was quantified in culture supernatants **(A)**. Expression levels of indicated genes and ALP activity are referred to DMSO cultures (dashed lines). Box plots were generated as described in Figure 1, outliers are depicted as ∘, and extreme values as □. *, *p* ≤ 0.05, Mann–Whitney *U* test. RUNX3 **(C)** and MEF2C **(D)** protein levels were determined in samples treated with IWP-2 (+) or with DMSO (–) by Western blot analysis, β-actin levels were determined as internal reference. Three representative experiments are shown.

Inhibition of canonical and non-canonical WNT-ligand secretion by IWP-2 during MPC chondrogenesis supports chondrogenesis while inhibiting hypertrophic and osteogenic marker expression, as described recently (Diederichs et al., 2019). When we specifically suppressed hypertrophy in MPC cultures by treatment with 2 μM IWP-2 from day 14 of chondrogenesis, *SOX9* and *COL2A1* remained unaffected, while *COL10A1* and *IBSP* expression was significantly reduced (Figure 7A). In parallel, ALP enzyme activity in the supernatant was significantly lower compared to controls. Remarkably, WNT inhibition during MPC chondrogenesis did not affect *RUNX2* and *RUNX3* levels, but reduced *MEF2C* gene expression significantly by 37% (Figure 7B). Western blot analysis confirmed that there was no regulation of RUNX3 protein (Figure 7C), while MEF2C protein levels declined when WNT signaling was inhibited (Figure 7D). Conclusively, only MEF2C, but not RUNX3, was driven by WNT signaling during MPC chondrogenesis.

### Treatment With PTHrP Pulses Reduce MEF2C Levels

**FIGURE 8:**
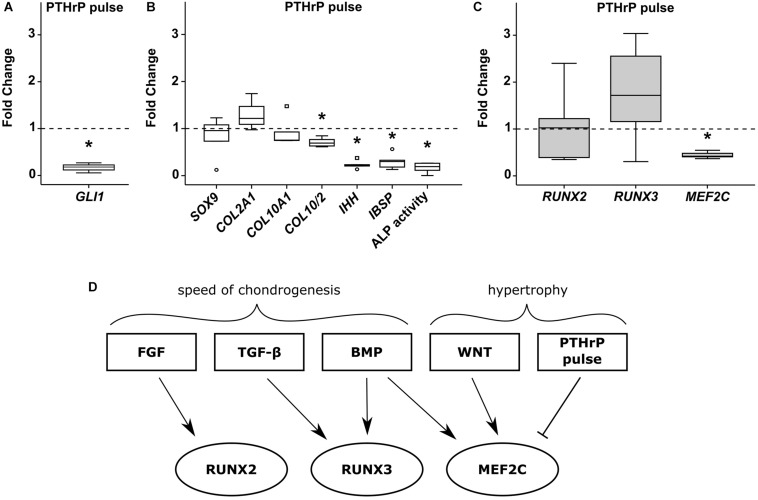
Effects of pulsed PTHrP treatment on RUNX2, RUNX3 and MEF2C. MPC pellets were subjected to chondrogenic induction for 1 week before half of the pellets were stimulated with 2.5 nM PTHrP(1-34) for 6 h daily while the others received daily medium exchange until week 6 (*n* = 5 donors). Expression of indicated genes was determined by qPCR **(A–C)**, and levels were referred to reference genes *HNRPH1* and *CPSF6*. **(A)** Gene expression of the HH downstream target *GLI1* (*n* = 3). ALP activity was quantified in culture supernatants **(B)**. Expression levels of indicated genes and ALP activity are referred to DMSO cultures (dashed lines). Box plots were generated as described in Figure 1, outliers are depicted as ∘ and extreme values as □. *, *p* ≤ 0.05, Mann–Whitney *U* test compared to control. **(D)** Overview of pathways regulating RUNX2, RUNX3, and MEF2C during MPC chondrogenesis.

Targeting of HH signaling during MPC chondrogenesis by intermittent stimulation of PTHrP signaling can reduce hypertrophy at maintained chondrogenic marker expression, as shown before (Fischer et al., 2016). When we treated MPC cultures from day 7 with PTHrP(1-34) for 6 h daily, HH signaling was reduced according to significant reduction of its downstream target *GLI1* (Figure 8A). While *SOX9* and *COL2A1* gene expression was maintained, the *COL10/COL2* ratio (−30%), *IHH* (−77%), and *IBSP* (−71%) expression levels were significantly reduced (Figure 8B). A strong suppression of ALP enzyme activity (−83%) compared to control pellets confirmed the anti-hypertrophic effect of this treatment (Figure 8B). Again, like with anti-hypertrophic WNT inhibition, PTHrP pulse treatment did not affect *RUNX2* or *RUNX3* expression levels (Figure 8C), while *MEF2C* expression was significantly lower at day 42.

In summary, each transcription factor was regulated by a unique set of pathways during MPC chondrogenesis (Figure 8D) with those affecting the speed of chondrogenesis (FGF-, TGF-β, and BMP-signaling) regulating *RUNX2*/*RUNX3* gene expression, while pathways driving hypertrophy at maintained chondrogenesis (WNT/HH-signaling) regulated only *MEF2C*.

### Functional Role of MEF2C and RUNX3

**FIGURE 9:**
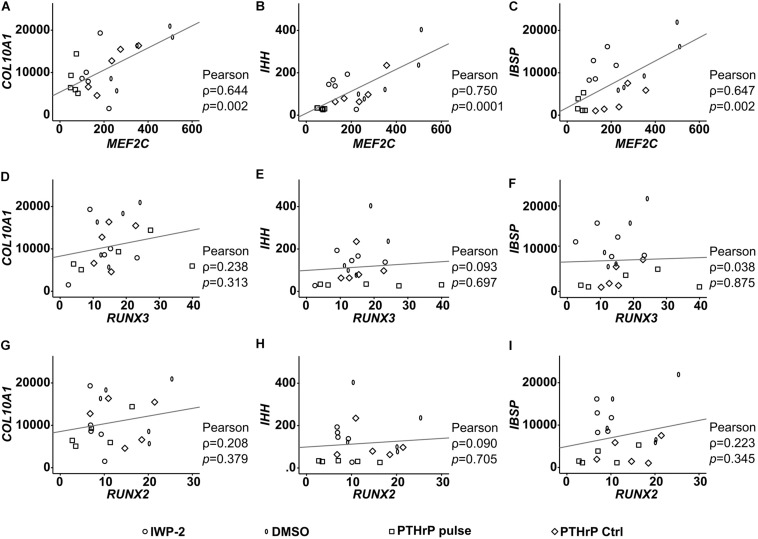
Correlation of RUNX2, RUNX3, and MEF2C with hypertrophic marker gene expression after lineage shift (WNT/HH inhibition). **(A–I)** Pearson correlation between the expression levels (% reference gene) of *RUNX2*, *RUNX3*, *MEF2C* and the hypertrophic genes *COL10A1*, *IHH*, and *IBSP* at indicated treatments, respectively.

**FIGURE 10:**
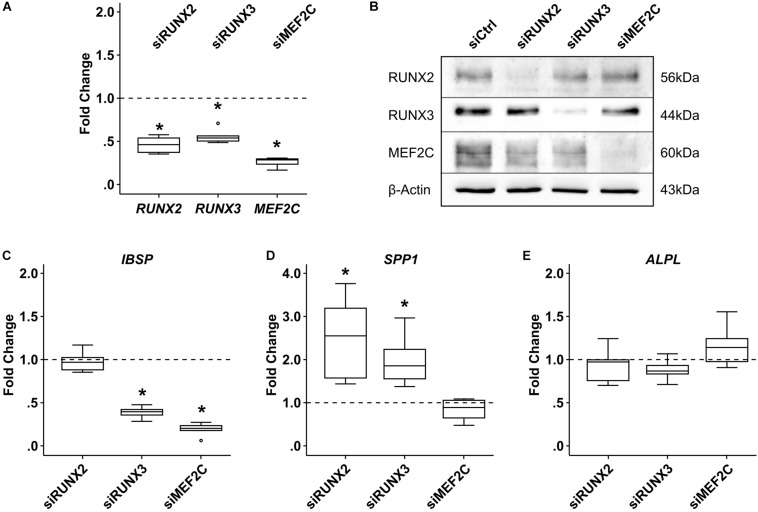
Effects of siRNA-mediated knockdown of MEF2C, RUNX2, and RUNX3 in SAOS-2 osteosarcoma cells. SAOS-2 cells were transfected with siMEF2C, siRUNX2, siRUNX3, or a non-targeting control siRNA (siCtrl) 48 h prior to analysis (*n* = 6 biological replicates from four independent experiments). **(A)** Target-gene expression was determined by qPCR. **(B)** MEF2C, RUNX2, and RUNX3 protein levels were determined by Western blot analysis; β-actin levels were determined as internal reference. One representative of four biological replicates (two to three experiments) is shown. Expression of indicated genes was determined by qPCR **(C–E)**, and levels were referred to reference genes *HNRPH1* and *CPSF6*. Expression levels of indicated genes are referred to siCtrl cultures (dashed lines). Box plots were generated as described in Figure 1; outliers are depicted as ∘. Significance compared to siCtrl was determined by Mann–Whitney *U* test *, *p* ≤ 0.05, with Bonferroni correction.

To establish a functional role for MEF2C and RUNX3 in hypertrophic marker expression, we next asked which hypertrophic/osteogenic markers typical for endochondral MPC differentiation may be downstream targets of these transcription factors. First, we performed correlation analyses between *MEF2C*, *RUNX3*, and *RUNX2* with the endochondral markers *COL10A1*, *IHH*, and *IBSP* in samples in which hypertrophy was specifically suppressed by manipulation of WNT or PTHrP/HH signaling. Importantly, only *MEF2C* levels correlated significantly with all three markers (Figure 9) while no correlation was obtained for *RUNX2* and *RUNX3*. Then, we performed specific siRNA-knockdown experiments in human MPCs subjected to chondrogenesis. Unfortunately, MPCs cannot be transfected during matrix-rich 3D pellet cultures, and lentiviral knockdown cannot be utilized, as it impairs the quality of MPC chondrogenesis, with control virus, demonstrating unspecific side effects of this technique in progenitor cells. Viral approaches allowing delayed inducible knockdown of transcription factors were performed but did not solve the issue. Since neither human tissue during endochondral ossification, nor human chondrogenic cell lines that undergo hypertrophic differentiation are available, we had to confine our knockdown analysis to the human osteosarcoma cell line SAOS-2, which expressed all three transcription factors on mRNA and protein levels. When *MEF2C*, *RUNX3*, and *RUNX2* were knocked down in SAOS-2 cells by a specific siRNA approach, a non-targeting control siRNA was included as a control. Strong and selective knockdown of the three transcription factors by the chosen siRNAs was confirmed on an mRNA as well as protein level (Figures 10A,B). While *IBSP* expression was significantly reduced by *RUNX3* and *MEF2C* knockdown, *IBSP* was not affected by RUNX2 suppression (Figure 10C). Intriguingly, osteopontin (*SPP1)* expression was even stimulated after *RUNX2* and *RUNX3* knockdown (Figure 10D), and *ALPL* expression remained unaffected by each single transcription factor knockdown (Figure 10E). Overall, the data suggested again no apparent role for RUNX2 for endochondral marker expression in human cells, while MEF2C and RUNX3 had *IBSP* as a confirmed downstream target and may, thus, drive *IBSP* expression levels in endochondral differentiation.

## Discussion

Runx2, Runx3, and Mef2c are the most important key fate-determining transcription factors directing cartilage development into the endochondral pathway in developmental mouse studies. Here, we investigated their expression, regulation, pathway dependence, and role during differentiation and hypertrophic chondrocyte specification of human MPCs. Higher *RUNX3* gene expression before the start of chondrogenesis and a rise of *RUNX3* and *MEF2C* mRNA expression parallel to hypertrophic markers were landmarks discriminating endochondral MPC development from chondral AC redifferentiation, which stimulated our interest into a functional connection between these transcription factors and endochondral outcome of cartilage neogenesis. Already, a closer look on protein levels questioned an active role for RUNX2 in driving MPC hypertrophy, since RUNX2 protein remained virtually absent, while RUNX3 and MEF2C proteins rose parallel to hypertrophy. Importantly, specific manipulation of chondrogenic pathways demonstrated that acceleration and deceleration of chondrogenesis modulated only the RUNX transcription factors, while specific suppression of hypertrophy, at maintained *COL2A1* expression, influenced only MEF2C. Strong and significant positive correlation of *MEF2C* levels with *COL10A1*, *IHH*, and *IBSP* expression levels, its unique regulation by the strongest anti-hypertrophic treatments at hand, and its functional role for upregulation of the osteogenic marker *IBSP* altogether suggest MEF2C as a valuable hypertrophic marker and the most promising new target to induce anti-hypertrophic effects in human cartilage neogenesis *in vitro*. Overall, our study unraveled a disparate regulation of RUNX2, RUNX3, and MEF2C during endochondral chondrocyte development of MPCs, connected a unique set of signaling pathways with upregulation of each factor and put MEF2C in the center of future studies to improve cartilage neogenesis from MPCs.

Runx2 and Runx3 are not essential for chondrocyte formation in mice, but are involved in early chondrocyte differentiation, and one or both of them must be present for endochondral maturation and vascular invasion into cartilage to occur (Yoshida et al., 2004; Komori, 2015). In mouse cartilage, Runx2 expression is higher than Runx3 expression, and therefore, Runx2 deficiency in mice results in more severe cartilage defects than a Runx3 deficiency. Thus, Runx2 appeared more important for hypertrophy of cartilage than Runx3 in mice (Yoshida et al., 2004). This may be the reason why, to date, all publications on human MPC chondrogenesis addressing RUNT transcription factors reported only on RUNX2 as a prohypertrophic factor, while RUNX3 has remained so far unaddressed (Mueller and Tuan, 2008; Cooke et al., 2011; Caron et al., 2013; Karl et al., 2014; Correa et al., 2015; Dexheimer et al., 2016; Fan et al., 2016; Fischer et al., 2016; De Kroon et al., 2017; Deng et al., 2018; Diederichs et al., 2019; Kim et al., 2019). Most of these studies analyzed *RUNX2* gene expression on a single (Cooke et al., 2011; Correa et al., 2015; Dexheimer et al., 2016; Fan et al., 2016; Fischer et al., 2016) or multiple timepoints during MPC chondrogenesis (Mueller and Tuan, 2008; Caron et al., 2013; Karl et al., 2014; De Kroon et al., 2017; Diederichs et al., 2019). However, the only group addressing *RUNX2* mRNA levels in weekly intervals during MPC chondrogenesis provided contradictive results, with significant upregulation on day 28 in one study (Mueller and Tuan, 2008), but not a follow-up study (Karl et al., 2014), and one study indicating a *RUNX2* stimulation by prohypertrophic conditions (Karl et al., 2014), and one study not (Mueller and Tuan, 2008). Thus, careful assessment of RUNX2 regulation on mRNA and protein levels, and assessment of RUNX3 and MEF2C during MPC chondrogenesis, is an important novelty of our study.

So far, only one group has visualized RUNX2 protein in human MPC chondrogenesis in photopolymerized hydrogels by Western blotting and suggested its downregulation under WNT pathway inhibition (Deng et al., 2018). Detection of high RUNX2 protein levels at start of chondrogenesis (Deng et al., 2018) is in apparent contradiction to our work. However, Deng et al. (2018) started MPC cultures, not from bone marrow aspirates but sieved cells from curetted and minced trabecular bone. By this, their cells expressed very strong RUNX2 protein levels after expansion, indicating a high osteoblast contamination of the used “MPC” population, which furthermore did not upregulate RUNX2 during chondrogenesis (Deng et al., 2018). We here started from virtually RUNX2-negative MPC populations, a profile which is typical for skeletal stem cell populations which did not yet undergo osteogenesis (Zouani et al., 2012). Our data on high quality MPC populations underline that RUNX2 protein never accumulated in human MPC chondrogenesis, except for some cells in the GAG-negative periphery of the pellet staining weakly positive by immunohistochemistry (Supplementary Figure S2). The fact that *RUNX2* mRNA levels did not rise significantly during chondrogenesis and had similar mRNA expression levels at days 28–42 of chondrogenesis-like SAOS-2 cells with their abundant RUNX2 protein levels in Western blots, indicates that the Western blot assay is sufficiently sensitive for RUNX2 protein detection. Prochondrogenic SOX9 protein degrades RUNX2 on the protein level (Zhou et al., 2006; Yamashita et al., 2009; Cheng and Genever, 2010) and, by this, attenuates its functions. We suggest that rising SOX9 protein during MPC chondrogenesis and its RUNX2-degrading capacity is one main reason why RUNX2 protein cannot accumulate during MPC chondrogenesis. Based on mRNA and protein data, we propose that RUNX3 and MEF2C, rather than RUNX2, are the more relevant transcription factors to be considered in the context of endochondral differentiation of human MPCs, since only RUNX3 and MEF2C were strongly upregulated, rose parallel to hypertrophic markers, and had the capacity to influence expression levels of the osteogenic downstream marker *IBSP* in SAOS-2 cells.

*In vitro* studies inducing chondrogenesis or osteogenesis in permanent mouse cell lines (Soung, Do et al., 2007; Bonyadi Rad et al., 2017) and *in vivo* developmental studies in mice described overlapping, cooperative, or redundant functions for Runx2 and Runx3 in endochondral differentiation (Stricker et al., 2002). However, details of their co-regulation and pathway-dependency are not well known in mouse and in human cells. Here, we used a well-established *in vitro* model of human endochondral chondrocyte differentiation, imitating many aspects of embryonal cartilage development (Dexheimer et al., 2012) and observed that *RUNX2* and RUNX3 remained unaffected by inhibition of WNT activity and pulsed PTHrP application. Remarkably, however, a disparate regulation was seen when the BMP and FGF pathways were modulated. BMP signaling stimulated only *RUNX3*, while in turn, FGFR signaling affected only *RUNX2*. FGFR inhibition during chondrogenesis reduced pERK1/2 activity and *RUNX2* expression (Figure 6), which is in line with the known induction of Runx2 by pERK1/2 activation in osteoblast differentiation (Wang et al., 2011). Furthermore, *Runx2* expression was elevated in mice carrying a mutant highly active FGF receptor1 (Zhou et al., 2000). Induction of only *RUNX3* at unaffected *RUNX2* was observed after BMP stimulation of the human gastric cancer cell line HT-29 (Lee et al., 2010), corroborating the non-redundant regulation of *RUNX2* and *RUNX3* expression by BMP signaling.

During embryonal cartilage development, FGF and BMP signaling are seen as two forces opposing each other (Niswander and Martin, 1993), and this is also observed in MPC chondrogenesis, where BMP pathway activation acts pro-chondrogenic (Hennig et al., 2007; Dexheimer et al., 2016), while stimulation of FGF signaling acts anti-chondrogenic (Weiss et al., 2010). Thus, it is interesting to note that *RUNX3* expression levels were here connected to pro-chondrogenic TGF-β and BMP signaling, while *RUNX2* mRNA levels were connected to anti-chondrogenic FGFR signaling. Given both transcription factors are functionally important, our data for the first time suggest different roles for RUNX2 and RUNX3 in specification of human MPCs for endochondral development. Further studies are needed to assess whether FGFR-driven RUNX2 may help to actively delay or stop chondrogenesis – for example, by repressing *COL2A1*, as one option to favor osteogenic lineage shift above chondrogenesis – while TGF-β/BMP-driven RUNX3 may actively promote osteochondral differentiation by upregulating hypertrophic/osteogenic markers as an alternate way to favor osteogenic marks. This would explain why RUNX2 failed to drive *IBSP* expression in SAOS-2 cells while RUNX3 did. Conclusively, specific regulation of RUNX2 and RUNX3 by opposite forces may, still, lead to the redundant outcome of a shift toward the osteogenic lineage, given SOX9 protein levels dropped sufficiently to allow RUNX2 accumulation as seen in the mouse growth plate (Lefebvre et al., 1998; Nishimura et al., 2012).

In mouse development Mef2c is a transcriptional activator of *Ihh* and *Col10aA1* expression in the growth plate which activates chondrocyte prehypertrophy from the Ihh-positive stage on. While Mef2c is sufficient to activate a full-length mouse collagen type X promoter, Runx2 is insufficient (Dy et al., 2012). In here, the currently most potent anti-hypertrophic treatments at our disposal, inhibition of WNT-signaling by IWP-2 and pulsed PTHrP treatment, which strongly suppressed *IHH* expression and reduced *COL10A1* without disturbing *COL2A1* expression, changed only MEF2C levels. This strongly argues that MEF2C is the most important pro-hypertrophic transcription factor during MPC chondrogenesis driving *IHH* and *COL10A1* expression. Further follow up, with human cells, is needed since transcription factor responsive elements in the *COL10A1* promoter differed between mouse and human (Higashikawa et al., 2009).

By conducting knockdown experiments in SAOS-2 cells, we here showed that MEF2C drives *IBSP* expression in line with reports in mouse MC3T3 cells (Stephens et al., 2011) and its pro-osteogenic role in this human osteosarcoma cell line. RUNX2 and RUNX3 knockdown in SAOS-2, however, painted a less-clear picture on the role of RUNX transcription factors for osteogenic marker regulation, since both suppressed osteopontin expression. Thus, we cannot rule out a non-causative co-regulation with hypertrophy or even some anti-hypertrophic functions for RUNX3, which may be concluded from the strong drop in RUNX3 protein after TGF-β withdrawal, coinciding with a strong rise of ALP activity, while MEF2C was unaffected. In gastric cancer cells, TGF-β-driven RUNX3 antagonized WNT-signaling via a RUNX3/TCF4/β-catenin complex (Lee et al., 2010), which in our model would result in suppression of hypertrophy (Diederichs et al., 2019).

Our study comes with several limitations. One limitation is that most of the primary cells used in this study were derived from patients with OA, and it cannot be excluded that this may have affected the phenotype of the cells. We harvested chondrocytes only from macroscopic intact cartilage areas and demonstrated by thorough characterization that redifferentiated ACs did not adopt any signs of OA-related hypertrophy that may originate from the tissue source. Also, there is no hint in the literature that, *vice versa*, healthy but not OA chondrocytes would express RUNX2, RUNX3, or MEF2C. Regarding MPCs, we and many other groups have documented that hypertrophy develops irrespective of whether the bone marrow aspirates were derived from OA patients or non-OA patients (Scharstuhl et al., 2007; Dexheimer et al., 2011; Brady et al., 2015). Nevertheless, unless data are repeated with cells from healthy donors, the OA background should be kept in mind.

A second limitation is the use of phenotypically abnormal osteosarcoma cells for knockdown studies in lack of a human model cell line closer to MPC chondrogenesis than SAOS-2 cells. Although we (Melnik et al., 2019) and others (Venkatesan et al., 2012; Frisch et al., 2014; De Kroon et al., 2017; Kim et al., 2019) successfully modified MPCs in monolayer culture by forced expression of transgenes, this approach is inapplicable in here, since chondrogenesis does not occur in 2D, but requires 3D culture. Importantly, our data demonstrate that microenvironmental cues like higher oxygen and/or better nutrient access were critical for MEF2C protein expression and ALP induction, while cells in the pellet center developed properly. This has important implications for the overexpression/depletion of transcription factors, which should be confined to the peripheral cells to allow proper functional conclusions in our model. Of course, strong promotors may be able to drive overexpression also in the pellet center, in spite of an inappropriate microenvironment, but this would not add to our understanding of the natural mechanisms of MPC hypertrophy we are searching for, since expression of downstream targets like IHH and IBSP may also depend on the richer microenvironment. Thus, we propose that further understanding of the here-described 3D phenomena of chondrocyte hypertrophy requires new models and techniques that combine forced target gene expression with exposure to critical microenvironmental cues like this is possible by layered cartilage tissue design and layered tissue mineralization in transwells (Kunisch et al., 2018).

## Conclusion

The unique regulation of RUNX2, RUNX3, and MEF2C by distinct sets of pathways argues in favor of the non-redundant roles of these transcription factors during MPC chondrogenesis. Virtual absence of RUNX2 protein with strong upregulation of RUNX3 and MEF2C in parallel to hypertrophic markers clearly place RUNX3 and MEF2C in the center of future research attention for establishment of their precise function in the mechanisms of endochondral chondrocyte development in human MPCs. Importantly, alteration of TGF-β, FGF, and BMP activity affected the speed of chondrogenesis together with RUNX2 or RUNX3 expression, while WNT inhibition and pulsed PTHrP application – the strongest anti-hypertrophic treatments at hand – influenced only MEF2C. Thus, accurate regulation of MEF2C and RUNX3 may be a key for optimal guidance of MPCs between chondral versus endochondral pathways to enable neogenesis of hypertrophy-resistant cartilage. This will bring MPCs closer to clinical translation as an alternate source for severely limited articular chondrocytes, which are resistant to hypertrophy.

## Data Availability Statement

The raw data supporting the conclusions of this article will be made available by the authors, without undue reservation, to any qualified researcher upon request.

## Ethics Statement

The studies involving human participants were reviewed and approved by the Ethikkommission der Medizinischen Fakultät Heidelberg. Written informed consent to participate in this study was provided by the participants’ legal guardian/next of kin or with informed written consent of the patient.

## Author Contributions

SID: conception and design, collection of data, data analysis and interpretation, manuscript writing, and final approval of the manuscript. JF: collection of data, data analysis and interpretation, and final approval of the manuscript. TW: provision of study material and final approval of the manuscript. SD: data analysis and interpretation, manuscript writing, and final approval of the manuscript. WR: conception and design, financial support, administrative support, data interpretation, manuscript writing, and final approval of the manuscript.

## Conflict of Interest

The authors declare that the research was conducted in the absence of any commercial or financial relationships that could be construed as a potential conflict of interest.
