# Supplementary material for: Significance of MEF2C and RUNX3 Regulation for Endochondral Differentiation of Human Mesenchymal Progenitor Cells
Source: Front Cell Dev Biol. 2020 Mar 4;8:81. doi: 10.3389/fcell.2020.00081 (PMC7064729; doi:10.3389/fcell.2020.00081)
Supplement: Supplementary file 1 [file Data_Sheet_1.pdf]

Supplementary Material

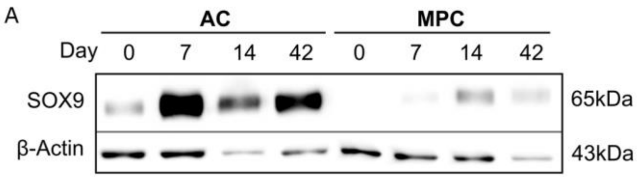

Fig. S 1 SOX9 protein regulation during AC redifferentiation vs MPC chondrogenesis. AC and MPC pellets were subjected to chondrogenic induction for 6 weeks and protein lysates prepared at weekly intervals. (A) Samples of 4 selected time-points were run on the same gel, and SOX9 protein levels determined by Western blot analysis using β-actin levels as internal reference (n=2).

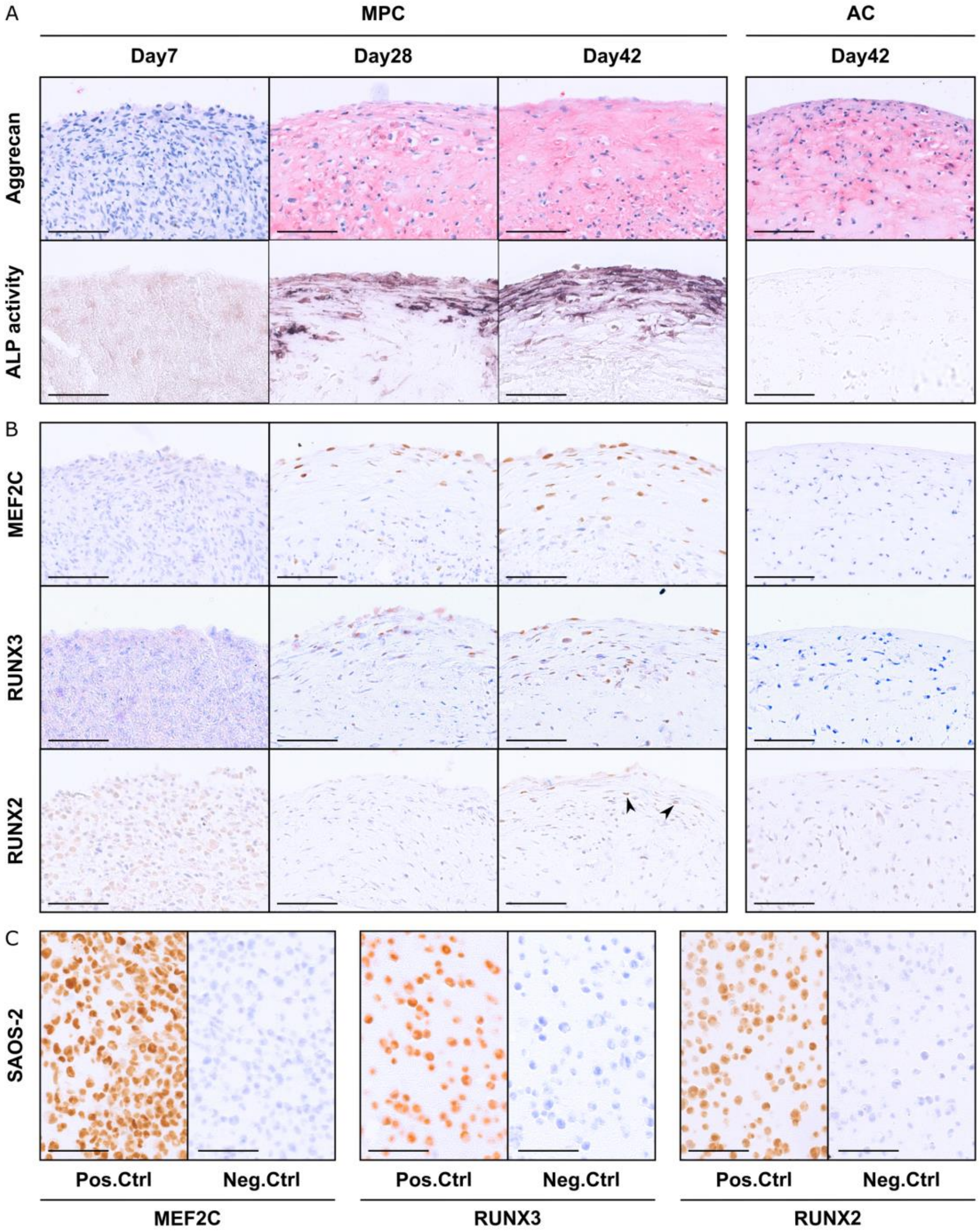

Fig. S 2 Histological detection of aggrecan, ALP activity, MEF2C, RUNX3 and RUNX2 during in vitro pellet culture. AC and MPC pellets were subjected to chondrogenic induction for up to 6 weeks and paraffin sections were stained for (A) aggrecan and ALP activity, (B) MEF2C, RUNX3 and RUNX2 at indicated timepoints. Arrowheads indicate cells weakly positive for RUNX2 (C) SAOS-2 cells embedded for 2h in fibrin hydrogel served as positive control for MEF2C, RUNX3 and RUNX2 staining, respectively. Negative controls: no primary antibody. Scale bars represent 100µm, n=2-5.
